# Selective expansion and differentiation of antigen-specific CD4^+^ T-helper cells by engineered extracellular vesicles

**DOI:** 10.1080/10717544.2025.2509969

**Published:** 2025-06-12

**Authors:** Ryouken Kimura, Tomoyoshi Yamano, Uryo Onishi, Xiabing Lyu, Kanto Nagamori, Toan Van Le, Mitsutoshi Nakada, Rikinari Hanayama

**Affiliations:** ^a^ Department of Immunology, Graduate School of Medical Sciences, Kanazawa University, Kanazawa, Japan; ^b^ Department of Neurosurgery, Graduate School of Medical Sciences, Kanazawa University, Kanazawa, Japan; ^c^ WPI Nano Life Science Institute (NanoLSI), Kanazawa University, Kanazawa, Japan

**Keywords:** Extracellular vesicle, cancer immunotherapy, targeted cytokine delivery, CD4+ T cells, selective expansion

## Abstract

Extracellular vesicles (EVs), particularly small EVs (sEVs), are lipid bilayer vesicles secreted by various cell types and play a key role in intercellular communication. These vesicles are promising tools for cancer immunotherapy owing to their biocompatibility, low immunogenicity, and capacity for targeted drug delivery. In this study, we aimed to assess the potential of engineered antigen-presenting EVs (AP-EVs) to selectively expand and differentiate antigen-specific CD4^+^ T cells. We engineered two types of AP-EVs: AP-EVs-Th1 expressing MHC class II, CD80, and interleukin (IL)-12 on their surface to promote Th1 differentiation, and AP-EVs-Th2 expressing MHC class II, CD80, and IL-4 to induce Th2 differentiation. *In vitro* experiments demonstrated that AP-EVs successfully induced the antigen-specific proliferation and differentiation of Th1 and Th2 cells, respectively. Notably, *in vivo* administration of AP-EVs-Th1 significantly enhanced the proliferation and differentiation of tumor antigen-specific Th1 cells, leading to robust anti-tumor effects in a murine melanoma model. These findings highlight the potential of AP-EVs-Th1 for cancer immunotherapy, particularly in augmenting CD4^+^ T cell responses. Furthermore, the versatility and adaptability of EV-based therapies make them beneficial for the development of personalized immunotherapeutic strategies for various cancer types, offering the advantages of targeted immune modulation, ease of use, and reduced risk compared to cell-based therapies.

## Graphical Abstract

**Figure:**
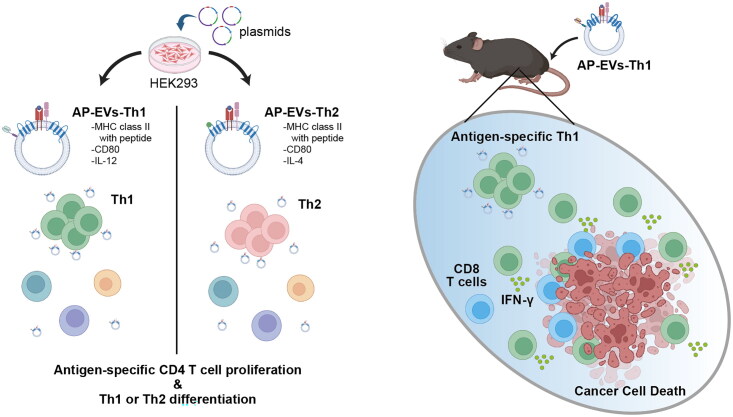

## Introduction

Small extracellular vesicles (sEVs), such as exosomes, are vesicular granules approximately 100–150 nm in size secreted by various cell types (Gao et al. 2021). sEVs are lipid bilayer vesicles that contain a variety of proteins, lipids, and nucleic acids reflective of their cell of origin. These components can induce functional and physiological changes in recipient cells (van Niel et al. 2018). For example, sEVs released by cancer cells or antigen-presenting cells have been reported to play a role in modulating immune response. Cancer-derived extracellular vesicles (EVs) express PD-L1, a ligand that binds to the inhibitory receptor PD-1 on T cells, thereby hindering the immune system’s ability to mount an effective anticancer response (Chen et al. 2018; Poggio et al. 2019). On the other hand, exosomes derived from mature dendritic cells (DC-sEVs) display peptide-major histocompatibility complex (pMHC), which can stimulate T cells either directly or indirectly (Pitt, André, et al. 2016; Lindenbergh and Stoorvogel 2018). In addition to their immunomodulatory potential, sEVs exhibit other characteristics that make them attractive for therapeutic applications, such as biocompatibility, low immunogenicity, and effective drug delivery capacity (Pitt, Kroemer, et al. 2016; Bonner and Willms 2021; Cheng and Hill 2022).

sEVs exhibit similar characteristics and biological activities as cell-based therapies for immunotherapy. Similar to cell-based therapies, sEVs specifically convey essential signals to the target immune cells. sEVs are safe alternatives to cell-based therapies for cancer immunotherapy owing to their cell-free nature, which reduces the risks associated with live-cell infusion, such as tumorigenicity and immune rejection (Giacobino et al. 2021; Janockova et al. 2021). Several preclinical studies have demonstrated the efficacy of sEVs for cancer immunotherapy (Yong et al. 2019; Giacobino et al. 2021; Ruan et al. 2022).

We previously demonstrated the efficacy of engineered antigen-presenting EVs (AP-EVs) in eliciting anti-tumor responses (Lyu et al. 2025). AP-EVs were designed to activate tumor antigen-specific CD8^+^ T cells via the surface expression of the peptide–major histocompatibility complex class I (pMHCI) complex, CD80, and interleukin (IL)-2 on EVs, resulting in significant anti-tumor activity. Selectivity of AP-EVs facilitates the targeted delivery of IL-2 to antigen-specific CD8^+^ T cells, thereby promoting their *in vivo* expansion. Therefore, AP-EVs are novel immunotherapeutic agents characterized by low side effects, simple formulation, and easy manageability, making them more advantageous than cell-based therapies.

In addition to the anti-tumor effects induced by activating CD8^+^ T cells, recent studies have also revealed the significance of CD4^+^ T cells in cancer immunity (Tay et al. 2021; Kruse et al. 2023). Alspach et al. reported that poorly immunogenic tumors engineered to express MHC II-restricted antigens induce Th1-polarized anti-tumor CD4^+^ T-cell responses and mediate long-term protection against subsequent tumor rechallenge in mice (Alspach et al. 2019). Yang et al. reported that CD4^+^ and CD8^+^ T cells transduced with a second-generation CD19-targeting chimeric antigen receptor (CAR) exhibit similar *in vitro* and *in vivo* efficacies in a mouse model of pre-B-cell acute lymphoblastic leukemia (Yang et al. 2017). CD4^+^ T cells expressing CAR are less susceptible to exhaustion than their CD8^+^ counterparts, highlighting the potential advantages of using CD4^+^ T cells in CAR T-cell therapy. Despite the growing importance of tumor immunotherapy using CD4^+^ T cells, the proliferation and differentiation of antigen-specific Th1 cells *in vivo* remain challenging (Constantino et al. 2017; Agliardi et al. 2021; Tay et al. 2021). Therefore, more practical approaches are urgently needed to regulate CD4^+^ T cell functions *in vivo*.

We hypothesized that engineered sEVs can be harnessed to modulate the immune responses of CD4^+^ T cells by expressing cytokines on their surfaces. Specifically, we postulated that these sEVs can aid in the control of the proliferation and differentiation of antigen-specific CD4^+^ T cells into Th1 and Th2 subsets. To verify this hypothesis, we engineered AP-EVs expressing cytokines conducive to the differentiation of Th1 and Th2 cells.

## Materials and methods

### Cell lines

Human embryonic kidney (HEK)-293 (CRL-1573; ATCC), retroviral packaging (PLAT-A; RV-102; Cell Biolabs, San Diego, CA), and ovalbumin (OVA)-expressing murine melanoma (derivative of B16; MO4; SCC420; Sigma-Aldrich, St. Louis, MO) cell lines were cultured in the Dulbecco’s modified Eagle’s medium (Thermo Fisher Scientific, Waltham, MA) supplemented with 10% heat-inactivated fetal calf serum (FCS; Gibco, Carlsbad, CA), 100 U/mL penicillin, and 100 U/mL streptomycin (Wako, Osaka, Japan). HT-2 cells were purchased from KAC Co., Ltd. (Kyoto, Japan) and cultured in RPMI-1640 medium (Thermo Fisher Scientific, Waltham, MA) supplemented with 10% heat-inactivated FCS, 0.05 mM 2-mercaptoethanol (2-ME; Gibco, Carlsbad, CA), and 100 IU/mL recombinant mouse IL-2 (BioLegend, San Diego, CA). All cell lines were cultured at 37 °C in a humidified atmosphere containing 5% CO_2_. To prepare EV-free FCS, FCS was mixed with PEG-10,000 (Merck, Kenilworth, NJ) at a 5:1 ratio and rotated at 4 °C for 3 h. Then, polyethylene glycol was removed by centrifuging at 2000 × *g* for 20 min, and the supernatant was passed through a 0.22-μm filter and used as EV-depleted FCS. AP-EV-producing cell line was cultured in the FreeStyle 293 Expression Medium (Gibco, Carlsbad, CA) supplemented with 50 U/mL penicillin and 50 U/mL streptomycin (FujifilmWako, Osaka, Japan).

### Mice

C57BL/6 mice were purchased from Japan SLC (Shizuoka, Japan) and housed in a specific pathogen-free (SPF) facility at Kanazawa University. OT-II T-cell receptor (TCR) transgenic mice were also maintained under SPF conditions (Hogquist et al. 1994).

### Preparation and purification of AP-EVs

To generate AP-EVs-Th1, HEK293 cells were engineered to stably express CD80–CD9, a fusion protein combining CD80 and tetraspanin CD9, and IL-12–milk fat globule-epidermal growth factor 8 (MFG-E8), a fusion protein linking the IL-12 single chain with MFG-E8 via a GGGGS linker, using retroviral systems. The cells are induced to transiently express IA-α and IA-β-CD81 using polyethyleneimine (Polysciences, Warrington, PA). To generate AP-EVs-Th2, HEK293 cells engineered to stably express CD80–CD9 were induced to transiently express IA-α and IA-β–CD81–IL-4 using polyethyleneimine. The cell culture supernatant was centrifuged at 300 × *g* for 5 min to remove the cell debris, at 1200 × *g* for 20 min to remove the cell debris and apoptotic bodies, and at 10,000 × *g* for 30 min to remove the apoptotic bodies and large EVs. AP-EVs were isolated from the supernatant via centrifugation at 100,000 × *g* for 4 h. The pellet was washed with phosphate-buffered saline (FujifilmWako, Osaka, Japan), and concentration of AP-EVs was quantified using the BCA assay (Thermo Fisher Scientific, Waltham, MA).

### Flow cytometric analysis of sEVs

Surface expression levels of MHC class II, CD80, IL-12, and IL-4 were analyzed using the PS Capture Exosome Flow Cytometry Kit (Fujifilm, Osaka, Japan), according to the manufacturer’s protocol. Briefly, 10,000 × *g* of supernatant was incubated with EV capture beads for 1 h, and EV-binding beads were stained with fluorescently labeled antibodies. After washing, the EV-binding beads were analyzed via flow cytometry (BD FACSCanto II, San Jose, CA).

### Nanoparticle tracking analysis

The number and size of AP-EVs were determined using a NanoSight LM10 (Malvern Panalytical, Malvern, UK). In brief, a 600 μL aliquot of the diluted AP-EV solution was introduced onto the analysis stage. Subsequently, the movement of AP-EVs was captured using a camera setting of 15 during a 30-second time frame. The analysis was performed by recording three distinct visual fields. The collected data was processed utilizing the NanoSight NTA software version 3.1 (Malvern Panalytical, Malvern, UK), with the detection threshold established at 3.

### Bioassay of IL-12 and IL-4

For IL-12 Bioassay, lymphocytes isolated from the spleens of C57BL/6 mice were plated in a 96-well plate at a density of 3 × 10^6^ cells per well. Serial dilutions of rIL-12, control EVs, and EVs expressing IL-12-MFG-E8 were then added to the respective wells. After 24 hours of incubation, the concentration of IFN-γ in the supernatant was quantified using the Mouse IFN-γ ELISA MAX™ Deluxe Set (BioLegend, San Diego, CA). For IL-4 bioassay, HT-2 cells were seeded in 96-well plates at 1 × 10^5^ cells/well, and serial dilutions of EVs that express CD81-IL-4 were added to each well. Cell viability was measured after three days of culturing using a WST-I colorimetric assay (Nacalai Tesque, Kyoto, Japan).

### Western blot analysis

1 × 10^8^ particles of EVs were lysed using a 2× SDS sample buffer composed of 100 mM Tris–HCl (pH 6.8), 4% (w/v) SDS, and 20% (v/v) glycerol, followed by separation through SDS-PAGE. The chemiluminescent signals from the Western blot were visualized using Luminata Western Chemiluminescent HRP Substrates (Merck Millipore, Kenilworth, NJ) and captured with the Fusion imaging system (Vilber, TechnoSaurus, Bern, Switzerland). The following primary antibodies were used for Western blot analysis: anti-human β-actin (Sigma-Aldrich, St. Louis, MO, clone: AC-15; 1:1000), anti-human CD81 (BioLegend, San Diego, CA, clone: 5A6; 1:1000), anti-mouse CD9 (Thermo Fisher Scientific, Waltham, MA, clone: EM-04; 1:1000), anti-MHC Class II (Thermo Fisher Scientific, Waltham, MA, clone: M5/114.15.2; 1:1000), and anti-mouse IL-12 p70 (Proteintech, Rosemont, IL, 1:1000). The secondary antibodies used were HRP Goat anti-mouse IgG (BioLegend, San Diego, CA, clone: Poly4053; 1:5000), HRP Goat anti-rat IgG (BioLegend, San Diego, CA, clone: Poly4054; 1:5000), and HRP Donkey anti-rabbit IgG (BioLegend, San Diego, CA, clone: Poly4054; 1:5000). Antibodies were diluted with Can Get Signals (TOYOBO, Osaka, Japan).

### Single EV analysis

EVs (4 × 10^9^ particles) were isolated by ultracentrifugation and labeled with fluorescence-conjugated antibodies at a 1:10 ratio in 40 µL of immobilizing buffer from the MagCapture™ Exosome Isolation Kit PS Ver.2 (FUJIFILM Wako Pure Chemical, Osaka, Japan) for two hours at room temperature. After labeling, the stained EVs were mixed with 960 µL of immobilizing buffer containing a binding enhancer. The antibody-labeled EVs were then purified by incubation with Tim4 conjugated beads. EVs were released from the beads by incubating the beads in 40 µL of elution buffer for 10 minutes, repeating the elution process twice. The isolated EVs were then subjected to analysis using a Flow Nanoanalyzer (NanoFCM, Xiamen, China) equipped with two lasers (488 nm and 638 nm) and three band-pass filters (488/10, 525/40, and 670/40). All measurements were carried out according to the manufacturer’s instructions. The obtained data were analyzed using FlowJo software version 10.4.1 (Ashland, OR).

### Electron microscopy analysis

AP-EVs were isolated using the ultracentrifugation method. The purified EVs, at a concentration of 5 × 10^10^ particles/mL, were transported to Hanaichi UltraStructure Research Institute located in Okazaki-shi, Japan for electron microscopy (EM) analysis. The EV samples were subjected to negative staining by placing a small aliquot of the sample onto a carbon-film grid and allowing it to settle for 10 seconds. Excess liquid was carefully removed, and a drop of 2% uranyl acetate staining solution was applied to the grid for an additional 10 seconds. Following this, the grid was blotted to remove excess stain and left to air-dry at ambient temperature. The negatively stained EV samples were then visualized using a JEOL JEM-1400Flash electron microscope (Akishima, Japan) operating at an accelerating voltage of 100 kV.

### **In vitro** T cell proliferation assay

Lymph node T cells isolated from C57BL/6 and OT-II transgenic mice were stained with 1 μM CellTrace Violet (CTV) Cell Proliferation Kit (Thermo Fisher Scientific, Waltham, MA) for flow cytometry and incubated at 37 °C for 3 min. The labeled cells were mixed at 1:1 ratio. Then, 1 × 10^5^ CTV-labeled mixed cells were co-cultured with 10 μg/mL of AP-EVs, control sEVs, or anti-mouse CD3/CD28 beads (Thermo Fisher Scientific, Waltham, MA) for three days. Subsequently, proliferation and differentiation of OT-II T cells were analyzed via flow cytometry.

### Adoptive T cell transfer

T cells from OT-II (CD45.1^−^2^+^) and C57BL/6 (CD45.1^+^2^−^) mice were mixed at 1:1 ratio and labeled with CTV. Then, 5 × 10^6^ labeled T cells were intravenously injected into recipient mice (CD45.1^+^2^+^). At one and four days post-injection, the recipient mice were subcutaneously administered 50 μg of AP-EVs-Th1 or control EVs. After three days, their spleen and lymph nodes were harvested, and single-cell suspensions were analyzed via flow cytometry.

### Antibodies and flow cytometry

Staining was performed according to standard procedures. The following monoclonal antibodies purchased from BioLegend (San Diego, CA) were used for extracellular staining: anti-CD4 (clone: GK1.5), anti-mouse CD45.1 (clone: A20), anti-mouse CD45.2 (clone: 104), anti-mouse MHC II, anti-mouse TCR-Vα2 (clone: B20.1), anti-mouse TCR-Vβ5.1,5.2 (clone: MR9-4), anti-mouse CD80 (clone: 16-10A1), anti-mouse IL-12 (clone: C15.6), and anti-mouse IL-4 (clone: 11B11). Intracellular cytokine staining was performed using antibodies against mouse interferon gamma (IFN-γ; clone: XMG1.2), T-bet (clone: 4B10), and GATA-3 (clone: 16E10A23) using the True-Nuclear Transcription Factor Buffer set (BioLegend, San Diego, CA). To determine the intracellular cytokine levels, cells were stimulated with 50 ng/mL of phorbol 12-myristate 13-acetate and 1 μg/mL of ionomycin for 6 h in the presence of 10 μg/mL brefeldin A. Flow cytometry data were acquired using the BD FACSDiva software and analyzed using FlowJo v.10.4.1 software (BD, San Jose, CA).

### Establishment of a tumor model

To establish the MO4 tumor model with adoptive OT-II T cell transfer, 1 × 10^5^ MO4 cells were subcutaneously injected into C57BL/6 mice. The next day, tumor-bearing mice were intravenously administered 5 × 10^6^ OT-II T cells. Subcutaneous injections of 50 μg AP-EVs-Th1 or control EVs were administered thrice, once every three days starting from day 1. Tumor volume was assessed every two days using a digital caliper and calculated as follows: length (mm) × width^2^ (mm^2^) × 0.5. Six mice each were randomly divided into the AP-EVs-Th1, control EVs, and non-treated groups. On day 10, approximately 100 μL of blood was collected from the tail vein of each mouse in the morning. Peripheral blood mononuclear cells were isolated via Ficoll density gradient centrifugation, and the percentages of TCR Vα2 and Vβ5 double-positive CD4^+^ T cells were determined via flow cytometry.

### Tumor-infiltrating lymphocyte (TIL) analysis

C57BL/6 mice were subcutaneously injected with 1 × 10^5^ MO4 cells. The next day, tumor-bearing mice were intravenously administered 5 × 10^6^ OT-II T cells. Two weeks after tumor inoculation, subcutaneous injections of 50 μg AP-EVs-Th1 or control EVs were administered twice, once every three days. The tumors were resected on day 21. The excised tumors were minced and suspended in the Dulbecco’s modified Eagle’s medium supplemented with 2 mg/mL Collagenase-D (Roche, Basel, Switzerland) and 100 μg/mL DNase (Roche, Basel, Switzerland). The tissues were incubated at 37 °C for 30 min with shaking, washed with fresh media, and passed through a 100-µm strainer to acquire a single-cell suspension. The tissue suspension was further incubated at 37 °C for 30 min with shaking, followed by washing with fresh media and passing through a 100 µm strainer to obtain a single-cell suspension. OT-II cell in TILs were gated on Zombie Aqua (to exclude the dead cells), CD4, Vα2, and Vβ5. Subsequently, the percentage of T-bet-expressing CD4^+^ T cells was determined via flow cytometry.

## Results

### AP-EVs express factors conducive to Th1 and Th2 differentiation

**Figure 1.**
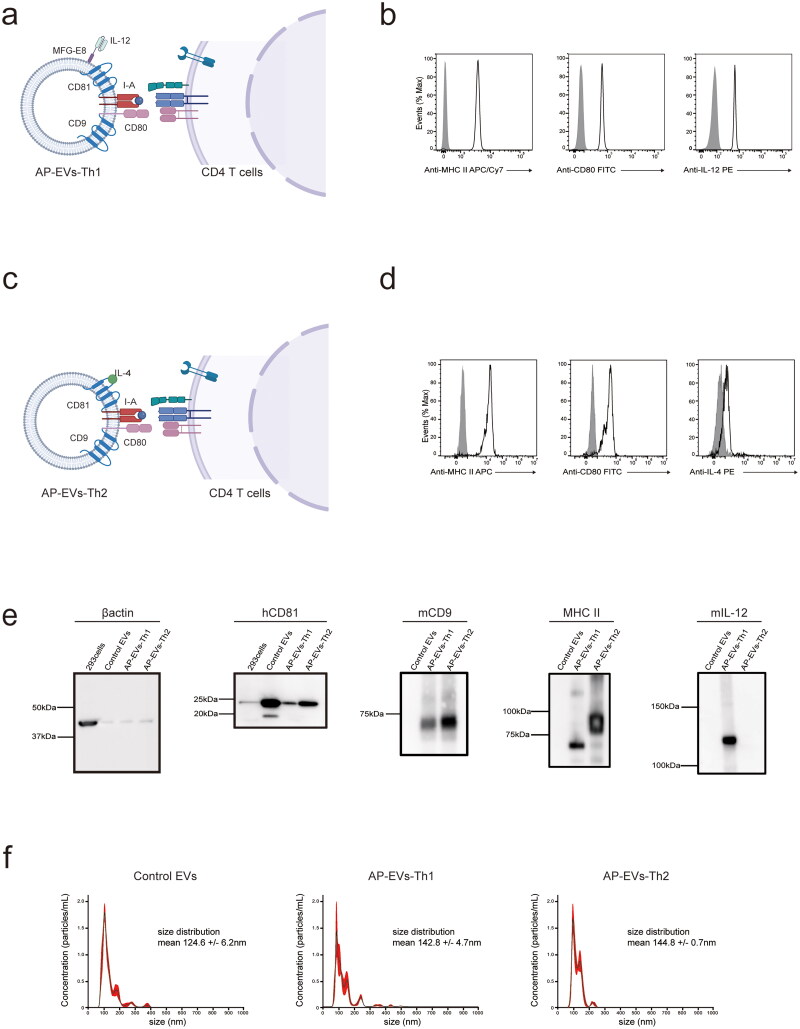
Establishment of antigen-presenting extracellular vesicles (AP-EVs). (a) Schematic representation of AP-EVs-Th1. Functional peptide–major histocompatibility complex class II (pMHCII) complex, CD80, and IL-12 interact with the T-cell receptor (TCR), CD28, and IL-12R, respectively, on antigen-specific CD4^+^ T cells. (b) Surface expression of MHC II, CD80, and IL-12 on AP-EVs-Th1. (c) Schematic representation of AP-EVs-Th2. Functional pMHCII complex, CD80, and IL-4 interact with TCR, CD28, and IL-4R, respectively, on antigen-specific CD4^+^ T cells. (d) Surface expression of MHC II, CD80, and IL-4 on AP-EVs-Th2. (e) Western blot analysis of AP-EVs. The blot confirms the presence of fusion proteins in AP-EVs and verifies the absence of cellular contaminants (e.g. human β-actin). (f) Nanoparticle tracking analysis histogram of AP-EVs. The histogram illustrates the size distribution of AP-EVs.

To investigate the role of engineered EVs in modulating T helper cell differentiation, we designed two types of EVs: AP-EVs-Th1 and AP-EVs-Th2. HEK293 cell line was transfected with four vectors to express the key proteins: (i) IA-α (α-chain of mouse MHC class II molecule), (ii) IAβ–CD81 (β-chain of mouse MHC class II molecule bound to the antigenic peptide of OVA through a GGGGS linker and tetraspanin CD81), (iii) CD80–CD9 (fusion protein of CD80 and tetraspanin CD9), and (iv) IL-12–MFG-E8 (fusion protein of recombinant IL-12 and MFG-E8) that binds to phosphatidylserine (PS) expressed on EVs (Andersen et al. 2000) (Figure 1(a); Fig. S1a–d). sEVs were purified from the culture supernatant via ultracentrifugation. Expression of these four proteins on sEVs was determined using flow cytometry (Figure 1(b)), and these sEVs were termed as AP-EVs-Th1. In AP-EVs-Th2, instead of recombinant IL-12–MFG-E8, IL-4, a key cytokine for Th2 differentiation, was inserted into the second extracellular loop of CD81 with a GGGGS linker, allowing for the robust and functional expression of IL-4 in these sEVs (Figure 1(c); Fig. S1a,c,e). Expression of the four proteins on the surface of AP-EVs-Th2 was also determined via flow cytometry (Figure 1(d)). The functionality of CD80 was evaluated by co-culturing OT-II cells with EVs expressing MHCII alone or co-expressing both MHCII and CD80 (Fig. S2a). The bioactivity of IL-12 and IL-4 on EVs was analyzed by either co-culturing EVs with splenocytes or HT-2 cells, the latter of which require IL-4 for survival (Fig. S2b and c). Western blot analysis validated the existence of fusion proteins within the AP-EVs while simultaneously confirming the lack of cellular contaminants, such as human β-actin (Figure 1(e)). To validate the co-expression of signals on AP-EVs, we confirmed the expression of MHCII–CD81–IL-4 and CD9–CD80 by NANO-FCM (Xiamen, China), a state-of-the-art flow cytometer that analyzes individual EVs, and found that over 50% of the EVs co-expressed MHCII and CD80 (Figure S2d). Nanoparticle tracking analysis revealed that the mean diameter of AP-EVs was approximately 140 nm, which was similar to the size of unmodified control EVs (Figure 1(f)). Transmission EM demonstrated that AP-EVs displayed the characteristic vesicular structure typically associated with EVs (Figure S2e).

### AP-EVs-Th1/Th2 induce OVA-specific proliferation and differentiation of Th1/Th2 cells

**Figure 2.**
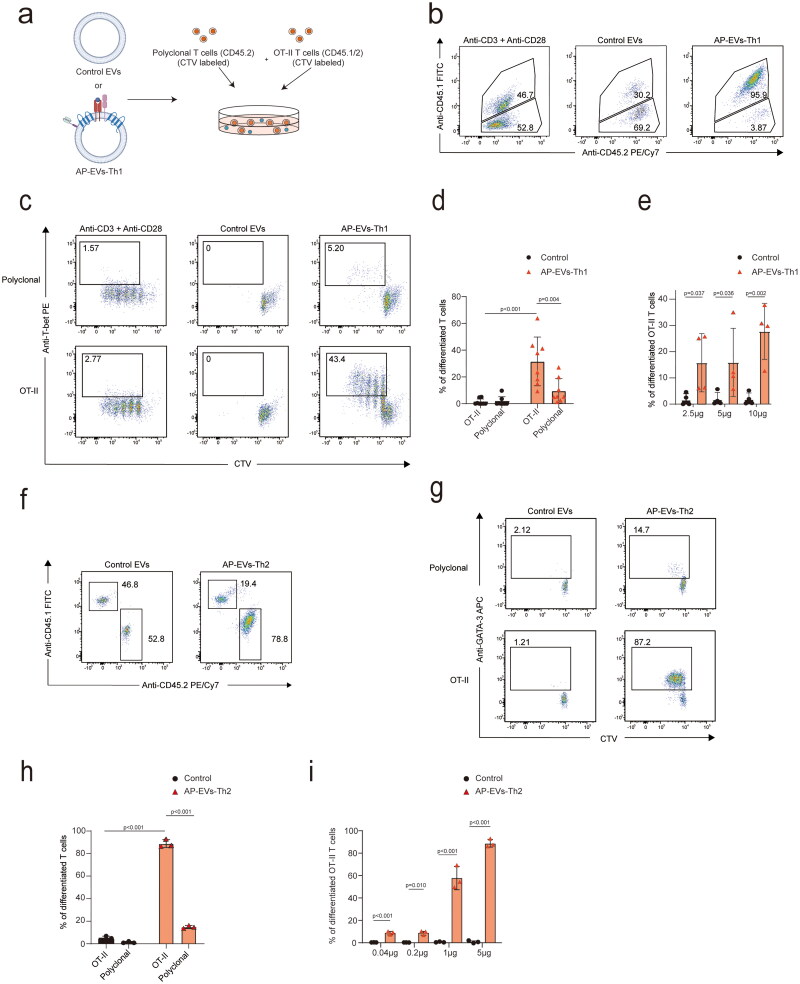
Selective expansion and differentiation of T cells by AP-EVs-Th1 and AP-EVs-Th2 *in vitro*. (a) Lymph node T cells from C57BL/6 and OT-II transgenic mice were combined in a 1:1 ratio. The combined cells were labeled with CellTrace Violet (CTV) and treated with control EVs or AP-EVs-Th1. (b) The combined cells were co-cultured with AP-EVs-Th1, control EVs, or anti-mouse CD3/CD28 conjugated beads for three days, and proliferation and differentiation of OT-II T cells were analyzed via flow cytometry. (c) Flow cytometric plots of CTV-labeled OT-II (CD45.2/CD45.1) and polyclonal (CD45.1) T cells cultured with anti-mouse CD3/CD28 conjugated beads, AP-EVs-Th1, or control EVs. (d) Percentage of T-bet-expressing CD4^+^ T cells. (e) Bar graph showing the percentage of T-bet-expressing wild-type (WT) CD4^+^ T or OT-II T cells under different concentrations of control EVs (black) and AP-EVs-Th1 (orange). (f) The combined cells were co-cultured with AP-EVs-Th2 or control EVs for three days, and the proliferation and differentiation of OT-II T cells were analyzed via flow cytometry. (g) Flow cytometric plots of CTV-labeled OT-II (CD45.2) and polyclonal (CD45.1) T cells cultured with anti-mouse CD3/CD28 conjugated beads, AP-EVs-Th2, or control EVs. (h) Percentage of GATA-3-expressing CD4^+^ T cells. (i) Bar graph showing the percentage of GATA-3-expressing WT CD4^+^ or OT-II T cells under different concentrations of control EVs (black) and AP-EVs-Th2 (orange).

Next, we investigated the effects of AP-EVs on the selective expansion and differentiation of Th1 and Th2 cells. Lymphocytes from C57BL/6 mice (CD45.1^−^2^+^) were mixed 1:1 with lymphocytes from OT-II mice (CD45.1^+^2^+^), which are transgenic mice expressing an OVA peptide-specific TCR, and labeled with CTV. Control EVs or AP-EVs-Th1 were added, and the cells were analyzed after five days. T cells stimulated with CD3 and CD28 antibodies exhibited nonspecific proliferation, and few T cells expressed T-bet, a master regulator of Th1 differentiation. In contrast, CD4^+^ T cells treated with AP-EVs-Th1 proliferated only in an OVA antigen-specific manner, and the proliferating T cells expressed T-bet, indicating antigen-specific Th1 cell differentiation by AP-EVs-Th1 (Figure 2(b–d)). The effects of AP-EVs-Th1 were concentration-dependent (Figure 2(e)). Similarly, we investigated the effects of AP-EVs-Th2 on the selective expansion and differentiation of Th2 cells. AP-EVs-Th2 induced the proliferation and differentiation of antigen-specific Th2 cells, as evidenced by GATA-3 expression (Figure 2(f–h)). The effects of AP-EVs-Th2 were also concentration-dependent (Figure 2(i)). We also confirmed that EVs, expressing only CD80, IL-12, or IL-4, did not induce the proliferation of OT-II T cells. In contrast, EVs expressing only MHCII induced T cell proliferation; however, both proliferation and differentiation of CD4^+^ T cells were weaker compared to those induced by AP-EVs-Th1 or AP-EVs-Th2. Although a mixture of EVs expressing single components showed some degree of proliferation and differentiation, the effect was less pronounced than that of AP-EVs-Th1 or AP-EVs-Th2 (Figure S3a and b). These data suggest that AP-EVs facilitate the selective expansion and differentiation of CD4^+^ T cells *in vitro*. By changing the cytokines expressed on AP-EVs, the differentiation of CD4^+^ T cells into Th1 or Th2 cells could be controlled, as indicated by the expression of their respective master regulators, T-bet and GATA-3.

### AP-EVs-Th1 induce the *in vivo* expansion of OVA-specific T cells and differentiation of Th1 cells

**Figure 3.**
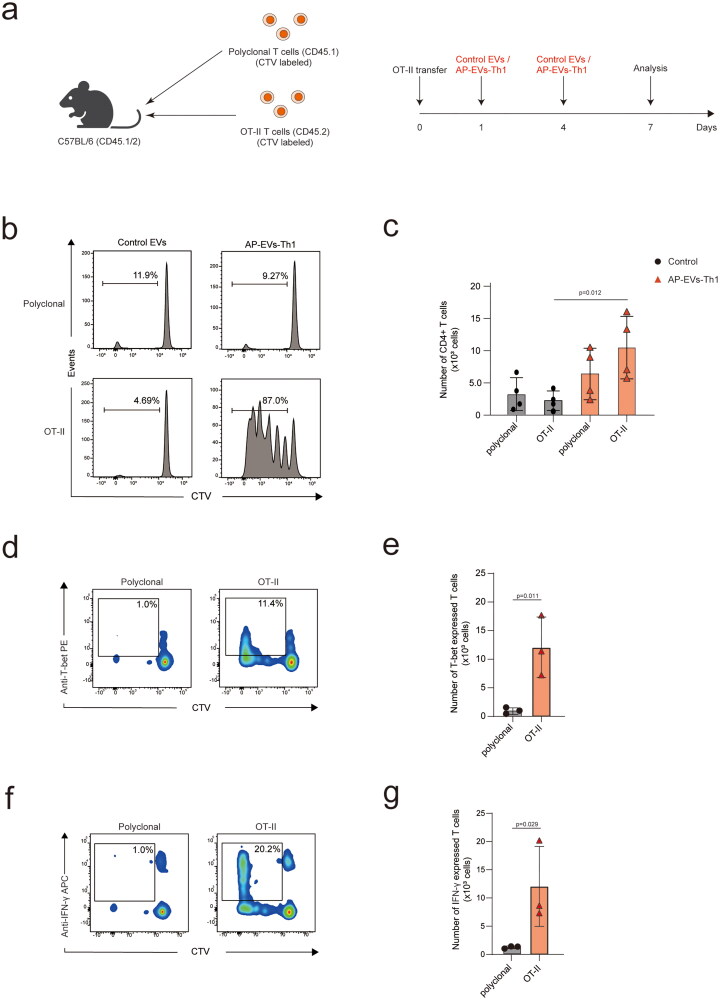
Selective expansion and differentiation of T cells induced by AP-EVs-Th1. (a) Experimental design. CTV-labeled lymph node T cells from C57BL/6 and OT-II transgenic mice (1:1 ratio) were injected into CD45.1^+^CD45.2^+^ recipient mice. The recipients were further subcutaneously injected with control EVs or AP-EVs-Th1 on days 1 and 4. (b, c) Spleens and lymph nodes were analyzed on day 7. (d, e) Proportion of T-bet^+^ cells in the spleen. (f, g) Proportion of IFN-γ^+^ cells in the spleen.

To explore the potential therapeutic applications of AP-EVs-Th1, we focused on Th1 cells, which exert anti-tumor effects (Haabeth et al. 2011; Xu 2014; Basu and Ghosh 2019). We investigated whether OVA-specific T-cell proliferation and Th1 differentiation can be induced *in vivo*. We transferred a mixture of congenitally marked OT-II (CD45.1^−^2^+^) and polyclonal (CD45.1^+^2^−^) T cells to the recipient mice (CD45.1^+^2^+^), subcutaneously injected control EVs or AP-EVs-Th1 on days 1 and 4, and analyzed CD4^+^ T cells on day 7 (Figure 3(a)). Control EVs did not stimulate cell proliferation, but AP-EVs-Th1 induced the proliferation of OVA-specific T cells (Figure 3(b,c)). Proliferating OT-II T cells expressed T-bet and IFN-γ, indicating that AP-EVs-Th1 differentiated OT-II T cells into Th1 cells (Figure 3(d–g)). These results suggest that AP-EVs-Th1 facilitate the selective expansion and differentiation of OT-II cells into Th1 cells *in vivo*.

### AP-EVs-Th1 exert anti-tumor effects

**Figure 4.**
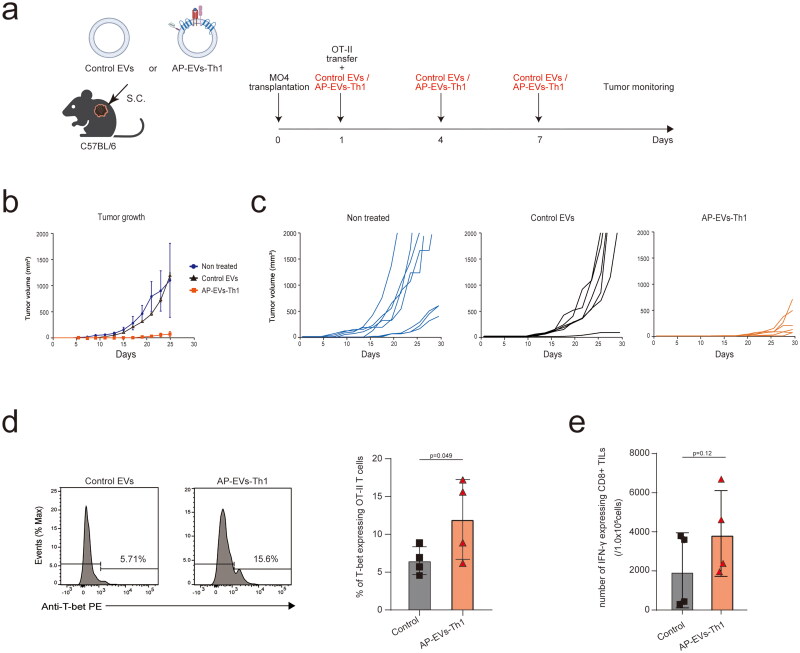
AP-EVs-Th1 exert anti-tumor effects on OT-II T cells. (a) Experimental design. OT-II T cells were administered to MO4 tumor-bearing mice, followed by subcutaneous injections of AP-EVs-Th1 or control EVs on days 1, 4, and 7. (b) Tumor growth curves indicate the untreated (blue), control EV-treated (black), and AP-EV-treated (red) mouse groups. (c) Flow cytometric analysis of tumor-infiltrating lymphocytes (TILs). (d) Percentage of T-bet + OT-II T cells in the tumor. (e) Number of IFN-γ^+^CD8^+^ cells in the tumor.

Finally, we investigated whether AP-EVs-Th1 exert anti-tumor effects by stimulating tumor-specific CD4^+^ T cell proliferation and differentiation. Recently, T cell receptor-engineered T cell (TCR-T) therapy, in which antigen-specific TCRs are introduced into T cells, has attracted attention (Schumacher 2002; Zhang and Wu 2018). Here, we conducted experiments in a mouse model to determine whether AP-EVs-Th1 enhance the efficacy of this therapy. Mice were inoculated with an OVA-overexpressing melanoma cell line, MO4. We transferred OT-II cells on day 1 and subcutaneously injected the mice with control EVs or AP-EVs-Th1 three times (Figure 4(a)). Notably, AP-EVs-Th1 were significantly more effective in reducing the tumor growth than the control EVs (Figure 4(b,c)). Analysis of OT-II T cells in TILs revealed a significantly higher percentage of CD4^+^ T cells expressing T-bet in the AP-EVs-Th1-treated group than in the control and non-treated groups (Figure 4(d)). Although not statistically significant, the number of IFN-γ-positive CD8^+^ T cells was increased in the AP-EVs-Th1-treated group (Figure 4(e)). These data suggest that AP-EVs-Th1 elicit anti-tumor effects via the *in vivo* proliferation and differentiation of tumor antigen-specific CD4^+^ T cells.

## Discussion

In this study, we successfully demonstrated the ability of engineered EVs to induce antigen-specific Th1 and Th2 cell differentiation. *In vivo*, antigen-specific Th1 differentiation enhances the anti-tumor efficacy. Application of IL-12, a key cytokine for Th1 differentiation, plays important roles in tumor immune responses (Trinchieri 2003; Knutson and Disis 2005). However, the application of IL-12 in cancer immunotherapy has been limited by its toxicity and moderate anti-tumor effects (Portielje et al. 2003; Lasek et al. 2014). Despite these limitations, IL-12 shows potential as an adjuvant for cancer vaccines and gene therapy (Lasek et al. 2016; Jia et al. 2022), suggesting that targeted and localized delivery of IL-12 could be a promising approach for cancer immunotherapy.

Exosome-based immunotherapy exhibits distinct features compared to cell-based immunotherapy. First, it demonstrates a high capacity to induce antigen-specific T cell differentiation and proliferation *in vivo*. By modifying the expressed components of AP-EVs to the intended profiles, AP-EVs can be used to promote the expansion and differentiation of both CD8^+^ and CD4^+^ T cells. This versatility of AP-EVs allows for the shaping of the desired immunological environment *in vivo*, highlighting the potential applications of combination therapies involving both CD8^+^ and CD4^+^ T cells. Second, exosome-based immunotherapy offers advantages in terms of simplicity of engineering. This conceptual framework enables the direct modification of antigens and cytokines via genetic manipulation. Unlike cell-based therapy, which requires meticulous management of culture media and cells, EVs can be conveniently stored under refrigeration once prepared. This inherent advantage can facilitate the off-the-shelf use of antigen-specific EVs in clinical practice.

Pharmacokinetics (PK) and pharmacodynamics (PD) analysis of EVs are critical for understanding their *in vivo* stability, biodistribution, and functional impact. Previous studies have shown that systemically administered EVs are rapidly cleared from circulation (Matsumoto et al. 2021), which suggests that the effects of AP-EVs observed in our study likely occur within a relatively short time frame. Future investigations incorporating detailed PK and PD studies will be essential to optimize the dosing regimen, administration route, and therapeutic efficacy of AP-EVs in cancer immunotherapy.

Potential applications of AP-EVs include their combination with other immunotherapeutic agents, such as engineered TCR-T cells (Schumacher 2002; Zhang and Wu 2018). By simultaneously activating the tumor antigen-specific CD8^+^ and CD4^+^ T cells, AP-EVs enhance the overall anti-tumor response. AP-EVs designed to promote Th1 differentiation can be administered with engineered TCRs targeting the same tumor antigen. This combination therapy exerts direct cytotoxic effects via engineered T cells and establishes a favorable immunological environment that supports and sustains the activity of these cells.

## Conclusions

In conclusion, this study demonstrated the potential of engineered EVs to induce antigen-specific Th1 and Th2 differentiation for immunotherapy. The ability of AP-EVs to induce antigen-specific cell differentiation and proliferation *in vivo*, combined with their easy application and management, makes them promising platforms for immunotherapy. Future studies should focus on overcoming the challenges of mass purification and human leukocyte antigen matching to facilitate the clinical applications of AP-EVs.

## Supplementary Material

Supplemental Material

## Data Availability

Upon reasonable request, data can be obtained from the corresponding author.
