# Supplementary material for: Selective expansion and differentiation of antigen-specific CD4+ T-helper cells by engineered extracellular vesicles
Source: Drug Deliv. 2025 Jun 12;32(1):2509969. doi: 10.1080/10717544.2025.2509969 (PMC12168395; doi:10.1080/10717544.2025.2509969)

**Supplementary material**

**Supplementary figure caption**

**Fig. S1.** Amino acid sequences of fusion proteins. Amino acid sequences of (a) I-Aα, (b) ovalbumin (OVA) peptide-I-Aβ–CD81, (c) CD80–CD9, (d) IL12– milk fat globule-epidermal growth factor 8 (MFG-E8), and (e) OVA peptide-I-Aβ–CD81–IL-4.


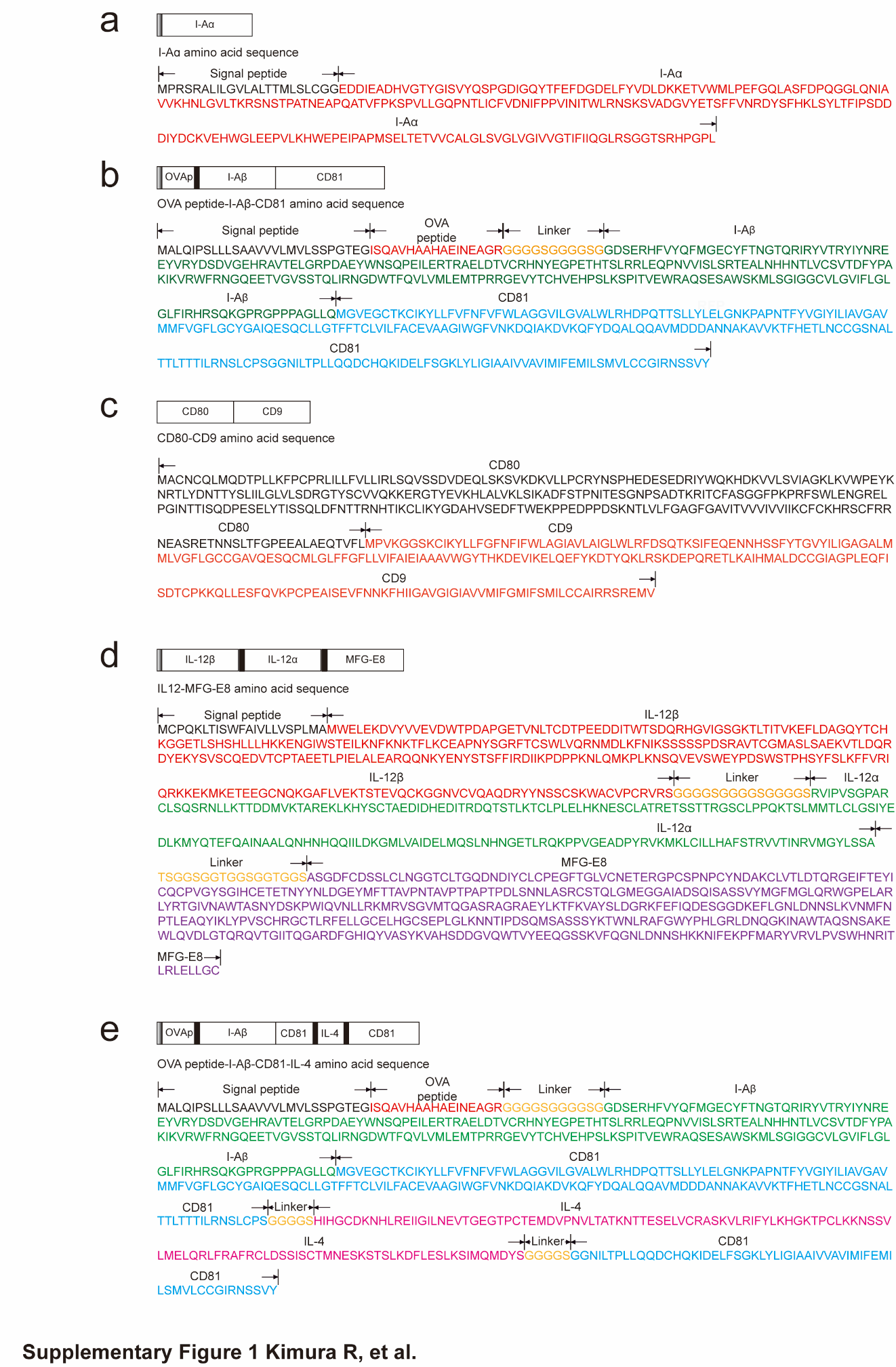


**Fig. S2.** Characteristics of AP-EVs. (a) The function of CD80 on EVs was analyzed by coculturing CTV-labeled OT-II T cells with EVs expressing either pMHCII or pMHCII and CD80. The left histogram represents CTV dilution of OT-II T cells cultured with MHCII expressing EVs. The right histogram represents CTV dilution of OT-II T cells cultured with MHCII and CD80 expressing EVs. (b) The function of IL-12 on EVs was measured by IL-12 bioassay using splenocytes. (c) The function of IL-4 on EVs was measured by IL-4 bioassay using HT-2 cells. (d) Nano-FCM analysis of AP-EVs. The left dot plot represents control EVs and the right dot plot represents AP-EVs-Th2. (e) Transmission electron microscopy image of AP-EVs. The scale bar represents 100 nm.


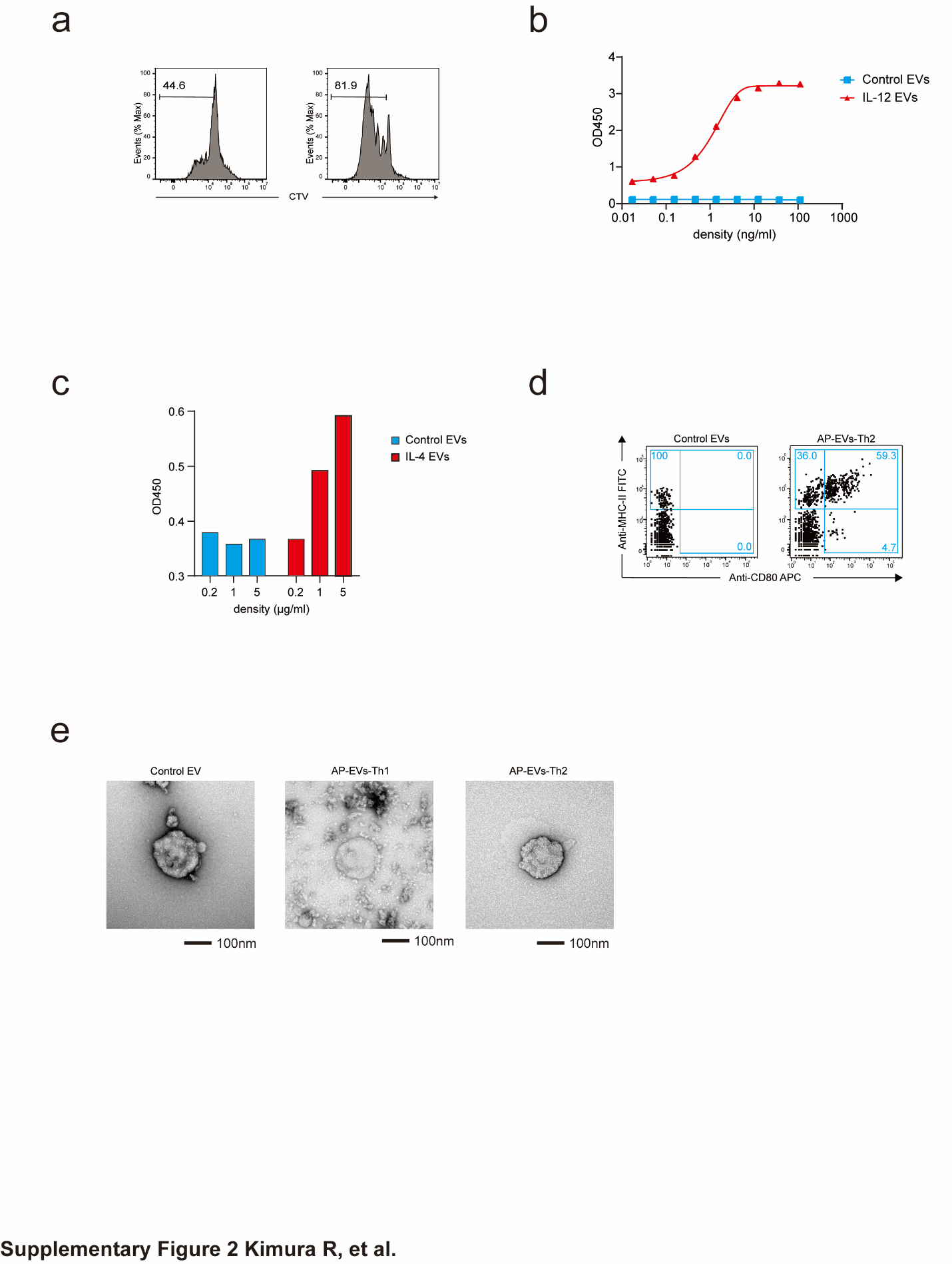


**Fig. S3.** The function of MHCII, CD80, and cytokines on EVs.

(a) 10μg of EVs expressing only a single component (MHCII, CD80, or IL-12) were cultured with CTV-labeled OT-II T cells for four days. In addition, 10μg of AP-EV-Th1 or 10μg mixture of EVs expressing MHCII, CD80, and IL-12 were cultured with CTV-labeled OT-II T cells for four days. (b) 10μg of EVs expressing only a single component (MHCII, CD80, or IL-4) were cultured with CTV-labeled OT-II T cells for four days. In addition, 10μg of AP-EV-Th2 or 10μg mixture of EVs expressing MHCII, CD80, and IL-4 were cultured with CTV-labeled OT-II T cells for four days.


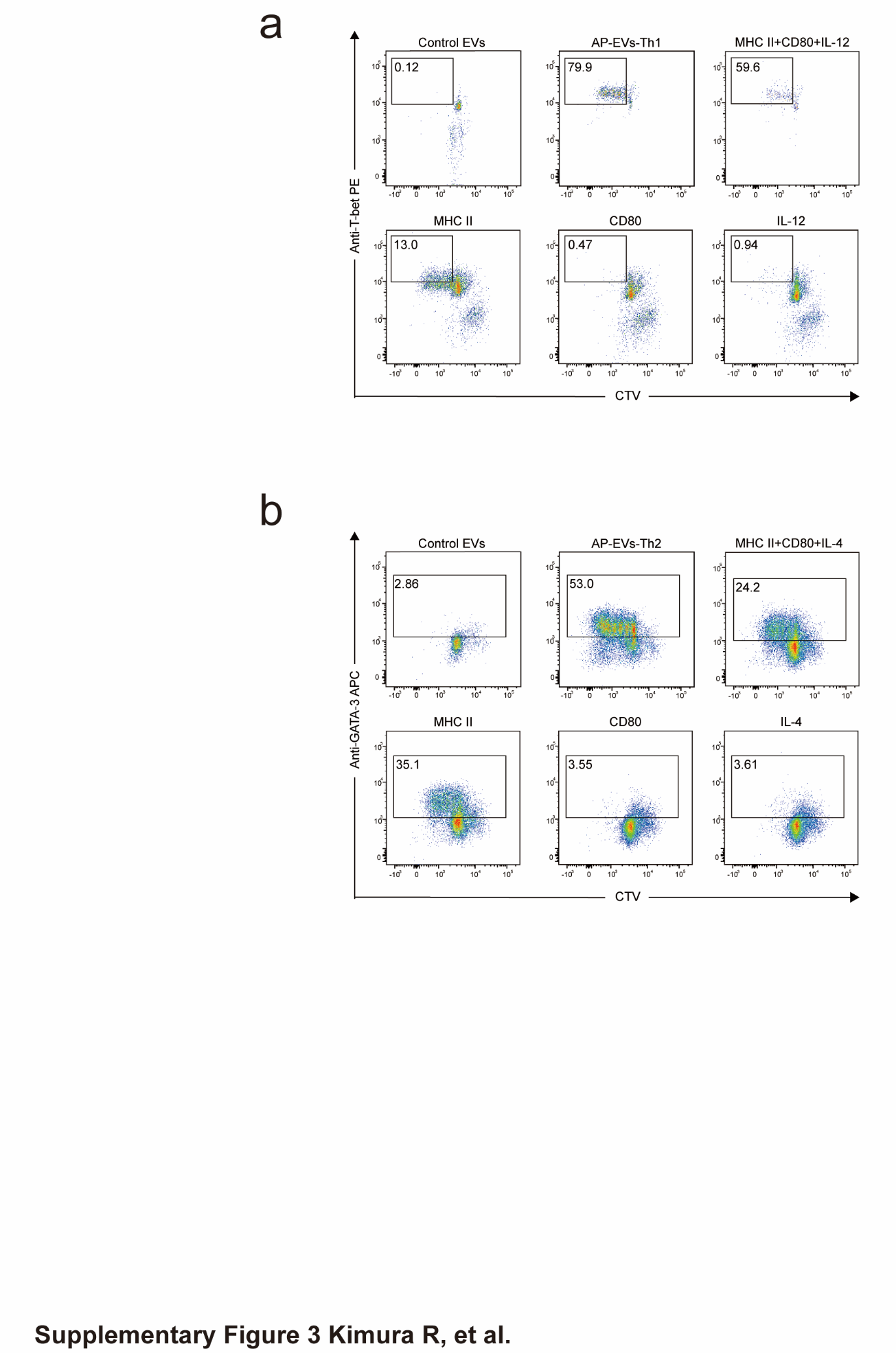

Supplement: Supplemental Material [file IDRD_A_2509969_SM9930.docx]
